# Why newly graduated nurses in South Korea leave their first job in a short time? A survival analysis

**DOI:** 10.1186/s12960-019-0397-x

**Published:** 2019-07-29

**Authors:** Eunhee Lee

**Affiliations:** School of Nursing/Research in Nursing Science, Hallym University, 1 Hallymdaehak-gil, Chuncheon, Gangwon-do 24252 South Korea

**Keywords:** Turnover, Job satisfaction, Working environment, Survival

## Abstract

**Background:**

South Korea is one of the countries with a very low percentage of active nurses among the Organization for Economic Cooperation and Development (OECD) countries. Although the number of nurses has increased steadily, the number of active nurses has not increased more than expected due to continued turnover.

**Methods:**

This study used data of a longitudinal panel of Graduates Occupational Mobility Survey (GOMS) and performed survival analysis to determine the turnover rate of nurses and the average time of turnover.

**Results:**

The turnover rate was the highest at 25% within first year and 50% of nurses left their first job during the study period. The hospital size and salary levels were major factors that affected the turnover rate, with small-scale hospitals and extremely low salary levels having the highest turnover. Dissatisfaction with the organization and dissatisfaction with the profession also directly impacted job turnover. Turnover rate of male nurses was higher than that of female nurses.

**Conclusion:**

Turnover of newly graduated nurses is highly inefficient personnel management. A strategy for reducing the turnover is needed.

## Introduction

Although nurse staffing level has a direct influence on patient outcome [1–3], the number of nursing staff is inadequate in South Korea. Of the Organization for Economic Cooperation and Development (OECD) countries, South Korea has the highest number of newly graduated nurses per 10,000 populations. However, the actual number of active nurses in South Korea is extremely low compared than that in other OECD countries [4]. Under this circumstance, the government has continually increased the entrance quota in nursing colleges. However, the number of active nurses has not increased significantly. As a result, in hospitalization care, nurses in South Korea are responsible for more patients per nurse than nurses in other countries.

Since the level of nursing staff is inadequate, the quality of nursing service as a whole is deteriorated and the safety of patient could not be guaranteed in the hospital. Such low level of nursing staff not only influences patients, but also affects nurses, resulting in dissatisfaction of nurses due to too much workload. Job dissatisfaction leads to a substantial turnover of nurses. An average of 16.9% of nurses has changed their job [5]. Among those who changed their job, the turnover of new nurses was approximately 30%, which was more than twice of existing nurses [5].

In order to increase the nursing staff level in South Korea, the government has introduced several policies to strengthen the staffing standard [6, 7]. However, some hospitals have difficulties to hire nurses. Hence, there is a problem in the supply and demand of the actual number of nursing personnel and the implementation of new policies. In the past, there have been many ways to increase nursing staff. One strategy was to increase the number of new nurses (increase the entrance quota in nursing college) [8]. Accordingly, the number of nurses in the nursing college was increased. It seems that it is no longer possible to solve the problem of nurse supply and demand by increasing nursing students since the number of new nurses per 10,000 populations in South Korea is the highest among OECD member countries. However, the number of active nurses in South Korea is still low [4]. In addition, an indiscreet increase of students may cause another problem such as quality deterioration of education.

Apart from increasing the number of students, a different approach is now needed. If medical institutions do not have enough nurses, it is difficult to expect a significant increase of active nurses even if there are plenty of nursing students due to their increase in the past decade. The recent strategy of non-active nurse re-employment also seems to be ineffective because the number of nurses who wish to be re-employed under current working conditions is currently unknown. Therefore, the most effective and prioritized strategy would be to maintain current active nurses.

Many cross-sectional studies on the dissatisfaction of nurses and nurses’ turnover intentions have been performed on a small scale. Nurses’ turnover is influenced by organizational factors and individual factors according to past studies. Individual factors include age, ability, dependents, sex, and education level while organizational factors include job satisfaction, burden in workload, stress, exhaustion, management style, empowerment, and role recognition [9–12]. However, most studies were focused on turnover intention and job satisfaction. Studies focusing on whether these factors lead to actual turnover are insufficient. In addition, it is difficult to determine whether turnover intention leads to actual turnover via cross-sectional studies. Furthermore, the high turnover intention may not lead to actual turnover. Therefore, factors that lead to actual turnover should be analyzed for actual nurse resource management. For these reasons, the objective of this study was to analyze factors affecting actual turnover rather than the turnover intention of newly graduated nurses.

## Methods

### Aim of the study

This study aims to analyze factors influencing the turnover of newly graduated nurses from university or college using data of Graduates Occupational Mobility Survey (GOMS), a longitudinal panel on nurses’ employment and turnover. The purpose of this study was to investigate factors affecting new nurses’ turnover.

### Data source and study subjects

The GOMS was conducted by South Korea Employment Informational Service. Its database had 3-year panel data of approximately 5% of graduates from college or university in South Korea by stratified random sampling. This longitudinal panel data was obtained through an initial survey next year after graduation with a followed up survey performed in 2 years after the initial survey. Data used in this study included 2008 GOMS, 2009 GOMS, and 2010 GOMS. Subjects of these data were graduates from 2008 to 2010. The initial survey was conducted in 2009 for 2008 graduates, 2010 for 2009 graduates and 2011 for 2010 graduates. The follow-up survey was conducted in 2011, 2012, and 2013 for 2008 graduates, 2009 graduates, and 2010 graduates, respectively.

This study only included the new nurses who started working as clinical nurses in hospitals after graduation from college or university. In South Korea, to qualify for the nurse license examination, there are two education system: diploma (3-year course) in college and degree (4-year course) in college or university. Since 2012, this education curriculum has been unified into a 4-year course regardless of the type of schools. In addition, the education system has incorporated a transfer system for students who would want to switch from other majors to nursing. Therefore, the subjects in this study include the graduates of a 3-year or 4-year course and the graduate students who have switched to the nursing course through the transfer system.

Since the objective of this study was to investigate the turnover of newly graduated nurses from their first job, the study subjects were those who started working as registered clinical nurses in hospitals. Therefore, department name, nursing, or nursing department was extracted from the database. Those who were employed in hospitals with occupations other than nurses as their first jobs were excluded. Since nurses could only work at the hospital after graduation and obtaining a license, those who started working at hospitals as nurses before graduation or before they became registered nurses or graduating a bachelor of science in nursing degree (BSN) program were excluded. The final sample of this study had a total of 652 students, including 174 graduates in 2008, 187 graduates in 2009, and 291 graduates in 2010.

### Variables

Explanatory variables used in the analysis were selected based on the findings of previous studies. These variables were classified into individual variables, organizational variables, and job satisfaction. Individual variables were gender, age, marital status, parental education level, family income (monthly), and education-related factors such as degree, admission type, and reasons for choosing a major. Organizational variables were extracted by hospital size, location, moving direction for employment after graduation, presence of labor union, salary, and shift. The hospital is currently classified into a semi-hospital, general hospital, and advanced general hospital in South Korea according to the bed size and hospital characteristics. In this study, the number of workers, not bed count, was used. Hospital in this study was classified into a small-scale hospital with less than 300 workers, medium-scale hospital with more than 300 workers and less than 1000 workers, and large-scale hospital with more than 1,000 workers based on a previous study [13]. Comparisons between locations of schools nurses graduated from and the workplace were made to find the moving direction of the workplace, analyzing whether they moved to the city, moved in the same area, or moved to a smaller city.

The survey of job satisfaction consisted of 12 items, including ten items on specific areas and two items on overall satisfaction with organization and profession. Satisfaction was measured on a 5-point scale. Graduated nurses were divided into two groups: dissatisfied group and satisfied group (satisfaction or normal).

### Analysis

Survival analysis was performed to analyze the turnover rate of nurses and the average time required for turnover. Kaplan-Meier survival curves of new nurses were used to determine the turnover rate. Differences in length of employment and the risk of factors affecting turnover were analyzed by Cox regression.

## Results

### Subject characteristics

Characteristics of 652 subjects are summarized in Table 1. Their average age at graduation was 24.8 years. The majority of subjects were females (90.5%) and unmarried (95.4%). Regarding parents’ education level, 33.4% and 16.9% of subjects whose father and mother were college graduates or more, respectively. Their average monthly income was 2–5 million Korean won (KRW). A total of 63.8% of participants graduated from college, which was more than the proportion of university graduates (36.2%). Most participants (93.6%) entered the nursing department as new students while 6.4% of them were transfer students. Regarding reasons for choosing nursing as their major, more than half (58.4%) of them chose nursing due to “job prospects” while 21.3% and 20.2% of them chose nursing due to aptitude and others as reasons, respectively. As for characteristics of medical institutions, the majority (51.4%) of them worked at large-scale hospitals, followed by 25.2% working at the small-scale hospital and 23.5% working at the medium-scale hospital. In terms of regional distribution, the majority (60.3%) of them worked at large cities including the capital. Many nurses were concentrated in Seoul. They considered it as the only area. Educational institutions are distributed evenly by region compared to medical institutions. The direction of choosing a job after graduation was mostly to the same area or in the direction of a big city. More than 50% responded that there was a labor union in their workplace and that they worked in a shift schedule. Regarding monthly salary, 70% of subjects had a monthly salary of less than 2.5 million won and 6.9% of them had a monthly salary of over 3 million won. Regarding job satisfaction scores for 10 factors, employment stability had the highest job satisfaction score, followed by social esteem and human relationship. Their overall satisfaction with the organization was found to be lower than that with the nursing profession.

**Table 1:** Individual factors and educational factors of newly graduated nurses (*n* = 652)

Variables	Categories	*N* (%) or M + SD [min–max]
Individual factor		
Graduation year	2008	174 (26.7)
	2009	187 (28.7)
	2010	291 (44.6)
Age at graduation [min–max]		24.8 ± 2.6 [22–46]
Gender	Female	590 (90.5)
	Male	62 (9.5)
Marital status	No	622 (95.4)
	Yes	30 (4.6)
Educational level (% of college or university)	Father	218 (33.4)
	Mother	110 (16.9)
Family income (monthly)	< 2 million won	152 (23.3)
	2–5 million won	389 (59.7)
	≥ 5 million won	111 (17.0)
Education factor		
Degree	College	416 (63.8)
	University	236 (36.2)
Admission type	New entrance	610 (93.6)
	Transfer	42 (6.4)
Reasons for choosing a major	Employment prospect	381 (58.4)
	Aptitude	139 (21.3)
	Others	132 (20.2)
Organizational factor		
Size	Small	164 (25.2)
	Medium	153 (23.5)
	Large	335 (51.4)
Location	Capital (Seoul)	210 (32.2)
	Metropolitan	183 (28.1)
	Non-metropolitan	259 (39.7)
Moving direction for employment	To the major cities	274 (42.0)
	In the same area	289 (44.3)
	To the minor cities	89 (13.7)
Union in the hospital	Yes	342 (52.5)
	No	310 (47.5)
Shift	Yes	387 (59.4)
	No	265 (40.6)
Salary (monthly)	< 2.0 million won	228 (35.0)
	2–2.5 million won	227 (34.8)
	2.5–3.0 million won	152 (23.3)
	≥ 3.0 million won	45 (6.9)
Job satisfaction (score 1–5)	Salary	3.21 ± 0.90
	Stability of employment	3.82 ± 0.81
	Working environment	3.25 ± 0.96
	Working hours	3.01 ± 1.00
	Career prospect	3.26 ± 0.87
	Relationship	3.41 ± 0.92
	Welfare benefit	3.36 ± 0.93
	Performance appraisal system	3.10 ± 0.80
	Social esteem	3.59 ± 0.82
	Autonomy and authority	3.20 ± 0.90
	Overall organization	3.16 ± 0.90
	Overall profession	3.28 ± 0.81

### Turnover of newly graduated nurses

**Fig. 1:**
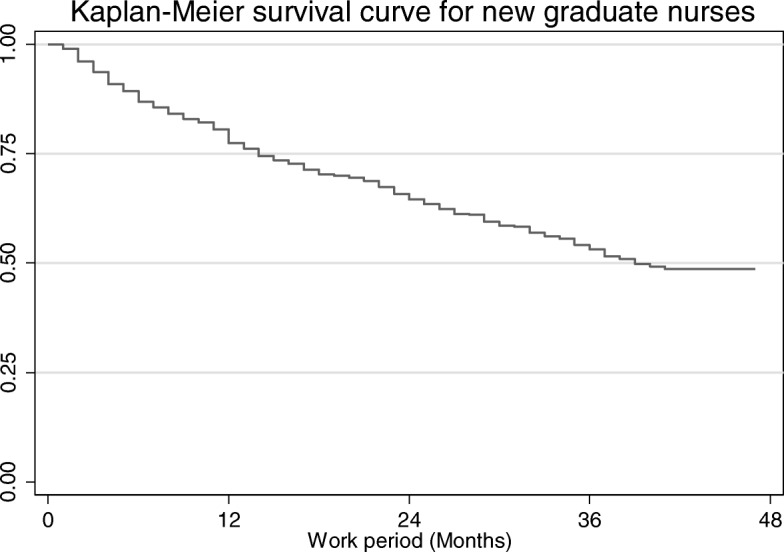
Turnover curve of newly graduated nurses

**Fig. 2:**
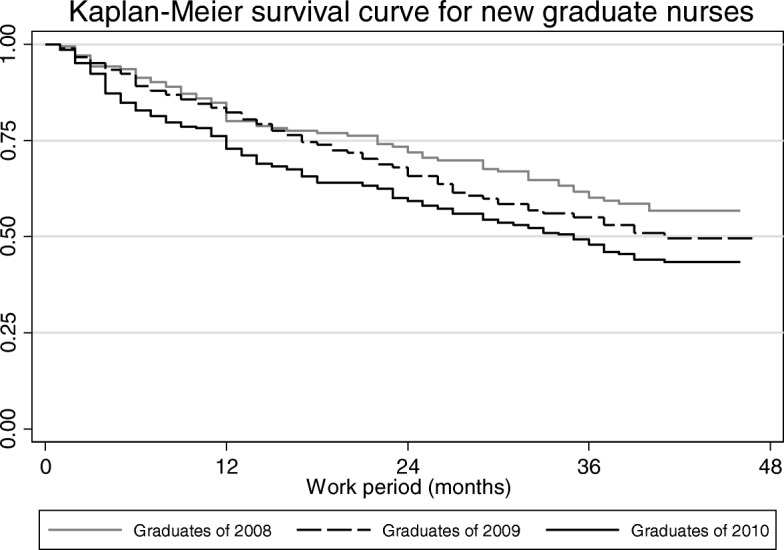
Turnover curve of newly graduated nurses by graduation year

For newly graduated nurses, turnover curve analysis showed that turnover within the first year was more rapid than that in the following period (Fig. 1). Within the first year, about 25% of employees changed their job. The number of unemployed nurses was continuously increased. Fifty percent of them had transferred their profession or organization. The annual turnover rate was gradually increased from 2008 to 2010. In particular, the turnover of nurses graduated in 2010 was higher than that in 2008 or 2009 (Fig. 2).

The results of the univariate analysis for investigating the factors affecting the turnover from their job showed in Table 2. Regarding turnover by individual factors, age was the only significant factor affecting turnover from their first job (HR 1.04, *p* = 0.022). The higher the age, the higher the risk of turnover. Among education factors, graduates from the university were less likely to leave their first job compared to the graduated from college. Among the hospital factors, the hospital size, union, and salary were the significant factors affecting the actual turnover. Newly graduated nurses were more likely to leave their first job when they worked in a small sized hospital in comparison to a hospital offering a lower salary or no union. Nonetheless, hospital size and salary were highly correlated variables affecting the turnover according to the interaction analyzed in univariate Cox regression. New nurses’ turnover was not affected by the salary level in a small size hospital and the hospital size at an extremely low salary level. Hence, the level of salary and the size of the hospital did not affect the turnover independently of each other. Results of satisfaction survey showed that the turnover was significantly higher when subjects were dissatisfied with their organization. Dissatisfaction with the organization was more closely related to job turnover than dissatisfaction with the profession itself. Nurses who were dissatisfied with most of these satisfaction factors were more likely to leave their first job. All factors showed statistically significant differences except salary, welfare benefit, and performance appraisal system. The risk of turnover for those having dissatisfaction with the organization was 2.74 times higher than those in the satisfied or normal group. In the case of profession satisfaction, the results also showed 2.09 times higher risk of dissatisfaction.

**Table 2:** Factors affecting the turnover of newly graduated nurses by univariate Cox regression analysis

Variables	Categories	Hazard ratio	*p* value	95% CI	
				Low	Upper
Individual factors					
Graduation year (ref: 2008)	2009	1.21	0.267	0.87	1.68
	2010	1.52**	< 0.001	1.14	2.03
Age at graduation		1.04*	0.022	1.01	1.08
Gender (ref: male)	Female	0.73	0.078	0.51	1.04
Marital status (ref: no)	Yes	1.24	0.397	0.75	2.06
Educational level of father (ref: less high school)	Father	1.04	0.751	0.82	1.33
	Mother	1.11	0.498	0.82	1.50
Family income (ref: < 2 million won monthly)	2–5 million won	0.88	0.352	0.67	1.15
	≥5 million won	0.80	0.236	0.56	1.16
Education factors					
Degree (ref: college)	University	0.77*	0.036	0.60	0.98
Admission type (ref: new entrance)	Transfer	1.39	0.129	0.91	2.13
Selection reason (ref: employment prospect)	Aptitude	0.94	0.688	0.70	1.26
	Others	1.04	0.813	0.77	1.39
Hospital factors					
Location (ref: capital)	Metropolitan	1.32	0.071	0.98	1.77
	Non-metropolitan	1.27	0.091	0.96	1.69
Moving direction for working (ref: to the major cities)	In the same area	1.21	0.138	0.94	1.55
	To the minor cities	1.36	0.082	0.96	1.91
Union (ref: no)	Yes	0.48**	< 0.001	0.38	0.61
Shift (ref: no)	Yes	1.30	0.096	0.95	1.77
Size (ref: small)	Medium	0.66**	0.008	0.49	0.90
	Large	0.51**	< 0.001	0.39	0.67
Salary (ref: < 2.0 million won)	2.0–2.5 million won	0.68**	0.004	0.53	0.88
	2.5–3.0 million won	0.34**	< 0.001	0.23	0.48
	≥ 3.0 million won	0.47**	0.004	0.28	0.79
Size and salary (ref: small, < 2.0 million won)	Small, 2.0–2.5 million won	0.73	0.140	0.48	1.11
	Small, 2.5–3.0 million won	0.39	0.188	0.09	1.59
	Small, ≥ 3.0 million won	1.63	0.629	0.23	11.78
	Medium, < 2.0 million won	0.81	0.333	0.52	1.25
	Medium, 2.0–2.5 million won	0.58*	0.012	0.38	0.89
	Medium, 2.5–3.0 million won	0.27**	0.001	0.12	0.59
	Medium, ≥ 3.0 million won^1^	–	–	.-	.-
	Large, < 2.0 million won	0.69	0.082	0.46	1.05
	Large, 2.0–2.5 million won	0.48**	< 0.001	0.32	0.72
	Large, 2.5–3.0 million won	0.28**	< 0.001	0.18	0.43
	Large, ≥ 3.0 million won	0.42**	0.003	0.24	0.74
Job dissatisfaction (ref: satisfaction or neutral)	Salary	1.25	0.102	0.96	1.63
	Stability of employment	3.01**	< 0.001	2.04	4.46
	Working environment	1.62**	< 0.001	1.25	2.08
	Working hours	1.39**	< 0.001	1.09	1.76
	Career prospect	1.55**	< 0.001	1.17	2.05
	Relationship	1.69**	< 0.001	1.26	2.28
	Welfare benefit	1.16	0.338	0.86	1.55
	Performance appraisal	1.05	0.737	0.79	1.40
	Social esteem	1.55*	0.026	1.05	2.27
	Autonomy and authority	1.47**	< 0.001	1.11	1.93
	Overall organization	2.74**	< 0.001	2.15	3.50
	Overall profession	2.09**	< 0.001	1.59	2.75

*p < 0.050, **p < 0.010, ^1^No turnover cases

The results of the multivariate analysis for variables identified in univariate analysis are shown in Table 3. After excluding mutually related variables, gender (male), small sized hospital, low salary, no union, dissatisfaction with the workplace, and dissatisfaction with the job influenced the turnover significantly from new graduated nurses’ first job.

**Table 3:** Factors affecting the turnover of newly graduated nurses by multivariate Cox regression analysis

Variables	Categories	Hazard ratio	*p* value	95% CI	
				Low	Upper
Graduation year (ref: 2008)	2009	1.37	0.069	0.94	1.85
	2010	1.75**	< 0.001	1.17	2.10
Gender (ref: male)	Female	0.66*	0.022	0.47	0.95
Size and salary (ref: small, < 2.0 million won)	Small, 2.0–2.5 million won	0.77	0.229	0.50	1.18
	Small, 2.5–3.0 million won	0.49	0.327	0.12	2.03
	Small, ≥ 3.0 million won	0.45	0.435	0.06	3.33
	Medium, < 2.0 million won	0.78	0.267	0.50	1.21
	Medium, 2.0–2.5 million won	0.62*	0.029	0.40	0.95
	Medium, 2.5–3.0 million won	0.24**	< 0.001	0.11	0.53
	Medium, ≥ 3.0 million won^1^	–	–	–	–
	Large, < 2.0 million won	0.68	0.077	0.45	1.04
	Large, 2.0–2.5 million won	0.49**	0.001	0.32	0.74
	Large, 2.5–3.0 million won	0.37**	< 0.001	0.23	0.59
	Large, ≥ 3.0 million won	0.63	0.130	0.35	1.14
Union (ref: no)	Yes	0.60**	< 0.001	0.47	0.77
Dissatisfaction	Organization	2.37**	< 0.001	1.76	3.20
(ref: satisfaction or neutral)	Profession	1.48^*^	0.021	1.06	2.06

*p < 0.050, **p < 0.010, ^1^No turnover cases

## Discussion

This study investigated the actual turnover of newly graduated nurses from their first job. During the study period, approximately 50% of newly graduated nurses left their first job while half of them (25%) left their first job within a year. Another study has reported that turnover in South Korea is 10.1–20.4% for all nurses and 27.8–33.0% for nurses with a career of less than 1 year [5, 14]. Considering these results, some nurses who left their first career within the study period might have moved to other hospitals. While the overall turnover rate in South Korea showed similar level with other countries [15–17], the rate of leaving their first workplace was quite higher than that in other studies [11, 15]. Although some nurses who left their first job might be employed again at other hospitals, organizations would have to spend additional cost for hiring and training new nurses due to skilled nurses’ turnover. Moreover, as a newly hired nurse cannot work as a skilled nurse in a short period of time, there is an additional nurse shortage in the hospital when the newly hired nurse is fully trained. Especially, there might be a serious problem in South Korea because the hospital in South Korea does not have enough labor force to deal with these situations. In South Korea, the number of nurses per population was extremely low among OECD member countries, although the number of beds was exceedingly high in South Korea [4]. Consequently, the low level of nurse staffing in hospitals might be a natural result under these circumstances. Therefore, frequent turnover in hospitals without having enough nurses can lead to a reduction in staffing level which can affect patients’ outcome [1, 18, 19].

This study included 2008 graduates, 2009 graduates, and 2010 graduates. The turnover showed a gradual increase in pattern. It showed a statistically significant difference according to the time of graduation. Graduates of 2010 particularly left their first job rapidly compared to graduates in 2008 or 2009. During 2008–2009, the turnover rate within a year was relatively low compared to that in 2010. The survival curve for graduates of 2008 showed a gradual decline. During that time, there were no changes in healthcare-related policies or demand and supply of health care workers in South Korea. This result might be partially attributed to the economic recession caused by the financial crisis in 2007–2008 [20]. Since entrance quota into the college of nursing in South Korea has steadily increased from 2008, the actual supply has started to increase from 2011 [8, 14]. Therefore, further research is necessary to investigate the effect of such increases on the nursing workforce.

In the univariate analysis, age, degree, union, hospital size, salary, interaction, and job dissatisfaction were factors influencing the turnover. Organizational factors rather than individual factors had an impact on the turnover. Several organizational factors affecting the turnover might have close relationships with each factor. For instance, nurses who work at small-scale organizations are underpaid compared to nurses who work at large-scale organizations [13, 21, 22]. In addition, small-scale organizations have a tendency not to have labor unions [23]. Workers’ salary in a hospital that has a labor union is higher than that in an organization without a labor union [23]. Hence, a hospital size, a union, and salary might be highly correlated with each other. In this study, the salary showed a linear pattern according to the hospital size. To analyze the impact of the interaction between hospital size and salary, the turnover according to the interaction term was analyzed in both univariate and multivariate analysis. Newly graduated nurses were more likely to leave a small sized hospital even though their salaries were high. These results indicated that salary is not a significant factor for turnover under vulnerable employment conditions. At the same time, at an extremely low salary, a hospital size has not affected the turnover from their first job. When the minimum conditions such as salary and benefit are satisfied, the turnover might be affected when the hospital is large and the salary level is high. Beyond salary and benefit, nurse staffing is inadequate in the small-scale hospital because their poor employment conditions make it difficult to hire nurses [13, 21]. Inadequate staffing exacerbates other working environments such as working hours and vacations. In this study, nurses who were dissatisfied with their working environment were more likely to leave their first job (HR = 1.62, *p* < 0.001), consistent with other research [24]. In addition to the working environment, turnover was increased when nurses were dissatisfied with the stability of employment or working hours. Hence, while the job itself is an important factor influencing nurses’ turnover, the working environment is also another major influencing factor. In a multivariate analysis in this study, turnover of the group dissatisfied with the workplace (HR = 2.37, *p* < 0.001) was higher than that of the group dissatisfied with work itself (HR = 1.48, *p* = 0.021). Beside organizational factor, satisfaction about professional itself such as career prospect, autonomy and authority, social esteem, and relation was also an influencing factor of turnover in this study. Hence, job satisfaction at both profession and organization is not less important than given circumstances such as hospital size or salary, consistent with the results of several studies [17, 24].

Among influencing factors, gender was the only significant factor in multivariate Cox regression. The turnover in male nurses was higher than that in female nurses, consistent with other studies [25]. This result might be partially attributed to gender imbalance because the nursing profession remains predominantly occupied by females. The proportion of male nurse in this study was also only 9.5%, similar to that in other countries [26]. There are problems arising from an extreme gender imbalance. The stereotype of women’s work hinders efforts to break such imbalance. Male nurses in South Korea also experience difficulties in adjusting in the organization with great gender imbalance [25, 27, 28]. To decrease the turnover of male nurses, further studies should investigate influencing factors overall and the difference according to gender.

This study has some limitations. First, not all variables derived from previous studies could be included because secondary data were used to investigate nurses’ turnover. In addition, turnover could not be divided into voluntary and involuntary due to the same reason as mentioned earlier.

## Conclusion

The lack of nurses is a severe problem in South Korea. We need to approach in more diverse ways to deal with this problem that many nurses are unable to work long after they are employed at hospitals. Regarding nurse turnover, quitting of newly graduated nurses is very inefficient for resource management in the view of an organization since there is no success compared to excessive input cost. Thus, medical institution and educational institution need to understand factors and strategies to reduce this turnover. Only gender in individual factors was a significant variable influencing nurse turnover in this study. Even if other variables were controlled, the risk of turnover for male nurses was higher than that for female ones. Further research is needed to determine what factors contribute to the turnover of male nurses as the proportion of male students in nursing colleges is gradually increasing. Organizational factors such as size, union status, and dissatisfaction with both organization and profession were factors that had major impacts on the turnover of nurses. The turnover is expected to be decreased if the work environment is improved. Factors related to job satisfaction need to be determined and strategies need to be developed to increase job satisfaction in the future.

## Data Availability

The data sets used and analyzed during this study can be provided from the corresponding author on reasonable request.

## Abbreviations

BSN: Bachelor of Science in Nursing degree
GOMS: Graduates occupational mobility survey
KRW: Korean won
OECD: the Organization for economic cooperation and development
